# PACAP Signalling Network in the Nucleus Accumbens Core Regulates Reinstatement Behaviour in Rat

**DOI:** 10.1111/adb.70090

**Published:** 2025-10-01

**Authors:** Samrat Bose, Gregory Simandl, Evan M. Hess, Linghai Kong, Nicholas J. Raddatz, Brian Maunze, SuJean Choi, David A. Baker

**Affiliations:** ^1^ Department of Biomedical Sciences Marquette University Milwaukee Wisconsin USA

**Keywords:** cocaine, dopamine D1‐like dopamine receptor agonists, dopamine D2‐like dopamine receptor agonists, nucleus accumbens, PACAP, reinstatement, self‐administration

## Abstract

Cocaine use disorder (CUD) lacks FDA‐approved treatments, partly due to the difficulty of creating therapeutics that target behaviour‐related neural circuits without disrupting signalling throughout the brain. One promising candidate for circuit‐selective neuromodulation is pituitary adenylate cyclase‐activating polypeptide (PACAP), a neuropeptide with pleiotropic behavioural actions whose signalling network spans the gut–brain axis. Here, we investigated the potential existence and function of an endogenous PACAP signalling network within the nucleus accumbens core (NAcc), which is a structure that integrates emotional, cognitive and reward processes underlying behaviour. We found that PACAP and its cognate receptor, PAC1R, are endogenously expressed in the rat NAcc and that PACAP mRNA is present in medial prefrontal cortical projections to the NAcc. Behaviourally, intra‐NAcc infusions of PACAP_1–38_ (450 ng/500 nL) did not induce seeking behaviour. Instead, it blocked cocaine‐primed reinstatement (10 mg/kg, intraperitoneal [ip]). Intra‐NAcc PACAP_1–38_ (450 ng/500 nL) also blocked reinstatement driven by coinfusion of the D1‐like dopamine receptor agonist (SKF81297, 3 μg/500 nL) but not the D2‐like dopamine receptor agonist (sumanirole, 100 ng/500 nL). These findings are notable because previous studies have shown D1‐like and D2‐like dopamine receptors in the NAcc regulate distinct processes and circuits. Collectively, these studies provide novel insights into the behaviour‐modulating actions of central PACAP signalling within the NAcc. Taken together with prior findings, the results underscore the need for additional research to reveal the precise behavioural processes and mechanisms that can be regulated by the full PACAP signalling network, which may reveal how to target this system to develop targeted therapeutics.

## Graphical abstract

This study demonstrates the presence of PACAP signalling in the nucleus accumbens, a key brain region for reward processing. In cocaine‐experienced rats, intra‐accumbal PACAP microinjection blocked cocaine‐induced reinstatement and prevented reinstatement driven by D1 but not D2 receptor activation, indicating circuit specificity. In the absence of FDA‐approved drugs for cocaine use disorder, PACAP emerges as a potential therapeutic candidate, although its precise mechanism of action needs to be investigated in detail.

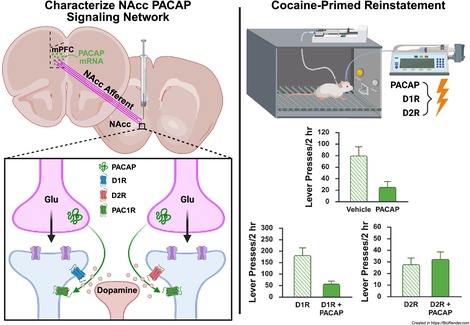

## 1 Introduction

Impaired behavioural control underlies chronic disorders such as addiction, stress‐related conditions and other psychiatric diseases, posing a significant public health challenge [1, 2]. Despite decades of research linking impaired behavioural control to dysfunction in neural circuits that integrate emotional, cognitive and reward processes [3, 4, 5, 6, 7, 8, 9], current treatment options remain limited. For example, cocaine use disorder (CUD) lacks a single FDA‐approved medication.

Developing therapies for central nervous system (CNS) disorders is far more challenging than for chronic diseases affecting other tissues [10, 11]. A key barrier to CNS therapeutic development is that many conventional experimental therapeutics act at neurotransmitter receptors, transporters or related mechanisms that are expressed by circuits distributed throughout the brain [12]. Hence, brain‐wide distribution of experimental therapeutics targeting conventional neurotransmitter systems will impact the activity of circuits that produce therapeutic outcomes as well as those that produce on‐target or off‐target adverse effects. Ultimately, the development of safe and effective treatments may require identifying novel strategies that effectively modulate behavioural control circuits selectively while avoiding broad CNS disruption.

The recent interest in peripheral–brain axis mechanisms has highlighted the therapeutic potential of utilizing peripheral signals that modulate central circuits involved in motivation and reward. Pituitary adenylate cyclase‐activating polypeptide (PACAP) is an endogenous neuropeptide from the secretin glucagon peptide family with both peripheral and central actions involving two isoforms, PACAP_1–27_ and PACAP_1–38_ [13, 14, 15], the latter of which is the most abundant isoform. Peripherally, PACAP released from the gut can influence brain function such as stress and hedonics by utilizing the gut–brain axis [16, 17, 18]. Additionally, PACAP exhibits a broad distribution in the CNS as it is expressed in cortical, subcortical and midbrain circuits [15]. Hence, central PACAP may be a key component of the gut–brain axis given the complex circuitry underlying CUD and related conditions [19, 20, 21, 22, 23, 24, 25, 26, 27].

Here, we investigated whether PACAP signalling in the nucleus accumbens core (NAcc) modulates cocaine‐seeking behaviour in rats following extinction from cocaine self‐administration, which has not previously been reported in the literature. We selected the NAcc given its role in integrating a diverse array of processes that regulate behaviour, including cognition, emotion and reward [3, 28, 29, 30, 31, 32]. First, we confirmed that PACAP_1–38_ and its canonical receptor, PAC1R, are endogenously expressed in the NAcc and that NAcc afferents from the medial prefrontal cortex (mPFC) express PACAP mRNA. We then found that intra‐NAcc PACAP_1–38_ dose‐dependently blocked cocaine‐primed reinstatement. Intra‐NAcc PACAP_1–38_ also suppressed reinstatement driven by intra‐NAcc coinfusion of the D1‐like dopamine receptor agonist SKF81297 but not by the D2‐like dopamine receptor agonist, sumanirole. Together, these results identify a previously uncharacterized role for intra‐NAcc PACAP signalling in regulating cocaine‐seeking behaviour and provide evidence for receptor‐specific modulation of dopaminergic reinstatement processes.

## 2 Materials and Methods

### 2.1 Animal Care and Usage

Male Sprague–Dawley rats (70–150 days) were individually housed in a temperature and humidity‐controlled room, maintained on a 12:12 light/dark cycle with lights off at 0700. Rats had ad libitum access to food and water, except during the self‐administration protocol. All procedures and protocols were approved by the Institutional Animal Care and Use Committee at Marquette University and adhered to the guidelines set forth by the National Institutes of Health.

### 2.2 Western Blotting

Rats were decapitated and 1‐mm^3^ NAcc tissue punches were lysed in 200 μL of ice‐cold lysis buffer (RIPA buffer with EDTA, Halt protease and phosphatase inhibitor cocktail; Pierce, Rockford, IL, USA). Protein extraction involved syringe homogenization (26‐gauge needle, 10 passes) and sonication (three pulses, 50% power) on ice. Homogenates were centrifuged (10 000 × *g*, 10 min, 4°C), and supernatants were collected. Protein levels were determined using a bicinchoninic acid (BCA) assay (#23252, Thermo Scientific, IL, USA). A total of 30‐μg protein was separated on 8% SDS‐PAGE and transferred to a polyvinylidene fluoride membrane. Membranes were blocked (5% BSA, 0.1% Tween‐20) and incubated overnight (4°C) with anti‐PAC1R antibody (1:1000, #AVR‐003, Alomone Labs, Jerusalem, Israel). After washing, membranes were incubated for 1 h at room temperature with an HRP‐conjugated anti‐rabbit secondary antibody (1:3000, #7074, Cell Signaling Technology, MA, USA). Bands were visualized using the Odyssey Fc Imaging System. Antibody specificity was confirmed by reprobing with and without a synthetic blocking PAC1R peptide (1:600, #BLP‐VR003, Alomone Labs, Jerusalem, Israel).

### 2.3 Microdialysis and PACAP Measurement

Using isoflurane anaesthesia and standard stereotaxic procedures, guide cannulae (22 gauge, Protech International, Roanoke, VA, USA) were surgically implanted in the NAcc (anterioposterior +1.2 mm, mediolateral ±2.4 mm and dorsoventral −6.7 mm with respect to bregma). Peptide‐recovery microdialysis probes (Eicom Atmos system, San Diego, CA, USA) with pressure‐cancelling ports and high‐molecular‐weight permeable membranes were inserted into the cannulas and perfused with dialysis buffer (1 μL/min) for 2 h. The probes were perfused with a dialysis buffer (153.5‐mM NaCl, 4.3‐mM KCl, 0.71‐mM CaCl_2_, 0.41‐mM MgCl_2_, 1.25‐mM glucose and 0.15% BSA) at 1 μL/min and stabilized for 2 h. Samples were then collected every 20 min for 2 h, pooled into a preservative solution (PBS, 0.5% Tween‐20, 2 × Halt Protease and Phosphatase Inhibitor Cocktail, ThermoScientific, Waltham, MA, USA) and analysed by ELISA (#MBS2516345, MyBioSource, CA, USA). Unstimulated extracellular PACAP levels ranged from 83 to 267 pM. Adjusting for probe recovery (< 10%), basal levels are estimated to be in the low‐nM range.

### 2.4 CTb Retrotracer Injection

Fluorescently conjugated cholera toxin subunit B (CTb 594, 1% dissolved in 1 × phosphate buffer; Invitrogen, CA, USA) was used to trace afferent projections to the NAcc. Using isoflurane anaesthesia and standard stereotaxic surgical procedures, 300 nL of CTb 594 was microinjected into the NAcc (32 gauge; anterioposterior +1.2 mm, mediolateral ±2.4 mm and dorsoventral −6.7 mm, at 7° with respect to bregma) at a rate of 50 nL/min. Animals recovered for at least 1 week prior to their inclusion in experiments.

### 2.5 In Situ Hybridization

Rats injected with CTb 594 in the NAcc were euthanized by rapid decapitation and brains were immediately frozen on dry ice. Brains were coronally sectioned on a cryostat at 12‐μm thickness, mounted onto electrostatically clean slides and stored at −80°C. Before hybridization, sections were postfixed in 4% paraformaldehyde, rinsed in 0.1‐M PBS (pH 7.4), equilibrated in 0.1‐M triethanolamine (pH 8.0) and acetylated in triethanolamine containing 0.25% acetic anhydride. Sense and antisense riboprobes targeting PACAP transcripts were generated via in vitro transcription, diluted in a hybridization cocktail (Amresco, Solon, OH, USA) with tRNA and hybridized overnight at 55°C with FITC‐labelled riboprobes. Slides were then treated with RNase A, washed in 0.1 × SSC at 65°C for 30 min and incubated overnight at 4°C with an antibody against FITC conjugated to horseradish peroxidase (HRP; Roche, Indianapolis, IN, USA). Riboprobe signals were amplified using the TSA‐Plus fluorophore system with fluorescein (PerkinElmer; Waltham, MA, USA). Images were acquired via confocal microscopy (Nikon Instruments Inc.; Melville, NY, USA) and analysed with NiS Elements software. FIJI (ImageJ 2) was used to reassign red fluorescence to magenta and overlay confocal images onto a standard rat brain atlas via the Big Warp Viewer plugin to visualize CTb 594 spread and PACAP mRNA coexpression.

### 2.6 Cannula Implantation for Drug Delivery to the Nucleus Accumbens

Bilateral guide cannulae (26 gauge) targeting the NAcc were implanted surgically in the brain under anaesthesia following the parameters mentioned earlier with stereotaxic coordinates (anterioposterior +1.2 mm, mediolateral ±2.4 mm and dorsoventral −4.7 mm, at 7° with respect to bregma). Cannulas were secured in position on the skull with screws and dental cement, with injectors extending 2 mm beyond cannula tips.

### 2.7 Jugular Catheter Surgery

Rats underwent surgical implantation of chronic indwelling jugular catheters terminating in the right atrium for cocaine self‐administration under isoflurane anaesthesia as mentioned earlier. A silicon‐tubing catheter (0.31‐mm inner diameter, 0.64‐mm outer diameter) was inserted into the right superior vena cava and sutured to the vein. Custom indwelling catheters were constructed of rounded tip polyurethane tubing (Access Technologies, Skokie, IL, USA) attached to a 22‐gauge back mount pedestal with attached mesh (Protech International, Roanoke, VA, USA). The exit port was positioned 2 cm posterior to the scapulae. Post‐surgical care included analgesia (meloxicam, 1 mg/kg, subcutaneous [sc]) and antibiotics (cefazolin, 100 mg/kg, intravenous [iv]) for pain and infection prevention, respectively. Catheters were flushed daily throughout the experiment duration with bacteriostatic heparinized saline to maintain patency and capped with Tygon tubing each time the leash/delivery line assembly was disconnected. Rats were allowed a 7‐day recovery period before the start of experimental procedures [33, 34].

### 2.8 Cocaine Self‐Administration Training

Rats were trained on a Fixed Ratio 1 (FR1) reinforcement schedule for daily 2‐h self‐administration sessions. Pressing the active lever extinguished the house light, activated a cue light and delivered either a sucrose pellet (food training) or a cocaine infusion (0.5 mg/kg/infusion, iv). Water was available ad libitum, but food was restricted to daily 20‐g rat chow post session. Initially, rats were trained to press for sucrose until achieving > 50 presses with < 15% variation over three consecutive sessions. During acquisition, training continued until rats received at least 12 daily infusions with < 15% variation across three sessions. Rats then progressed to maintenance under long‐access conditions (1.0 mg/kg/infusion; 6 h/day for 12 sessions), followed by a 7‐ to 10‐day withdrawal period during which bilateral guide cannulas were implanted, and rats were allowed to recover before extinction training. During extinction, pressing the active lever activated the cue light and syringe pump but delivered no infusion. Extinction criteria were set at two consecutive days of ≤ 15 lever presses, followed by a mock injection. If the mock injection induced > 15 responses, extinction criteria were reset, and an additional mock injection was performed to confirm stability.

### 2.9 Reinstatement Testing

Rats received 10‐mg/kg cocaine (intraperitoneal [ip]) immediately prior to testing. Pharmacological interventions included bilateral intracranial microinfusions of vehicle (0.9% saline), PACAP_1–38_ (225, 450 ng/500 nL, #350‐35, Echelon Biosciences, UT, USA), SKF81297 (3 μg/500 nL, #1447, Tocris, Bristol, UK) in 16% DMSO or sumanirole (100 ng/500 nL, #2773, Tocris, Bristol) at 125 nL/min in 16% DMSO 10 min before testing, with lever pressing recorded for 2 h. All DMSO solutions were prepared in 0.9% saline. Animals underwent a maximum of two reinstatement tests, with the second test conducted only after re‐establishing extinction criteria (≤ 15 presses on two consecutive extinction days).

### 2.10 Histology

At the completion of self‐administration experiments, rats were perfused with saline followed by 4% paraformaldehyde for fixation. The brains were sectioned into 150‐μm slices using a Vibratome, stained with cresyl violet and imaged. Brain images were aligned to a rat brain atlas (Paxinos and Watson, sixth edition) to confirm probe placement for data inclusion [35].

### 2.11 Statistical Analysis

Individuals conducting behavioural experiments were blinded to pharmacological interventions. Results were expressed as means with standard error of the mean (*M* ± SEM), accompanied by individual data points for independent samples. Graphs were generated using GraphPad Prism 11 (Dotmatics, Boston, MA, USA). Statistical analyses were performed using SPSS v29.0.0.0 (IBM, Armonk, NY, USA). A paired *t* test compared means between two related groups, and one‐way ANOVAs compared means across three or more groups. The Holm–Sidak procedure controlled the familywise *α* level at 0.05 for multiple post hoc comparisons. Effect sizes were calculated using partial eta‐squared (*ηp* ^2^) for ANOVA main effects and interactions and Cohen's *d* for planned pairwise comparisons, including Student's *t* tests. Analytical details unique to each experiment, including the number of rats, degrees of freedom and follow‐up statistical analysis, are reported in the corresponding results section.

## 3 Results

### 3.1 Existence of a PACAP Signalling Network in the NAcc

**FIGURE 1:**
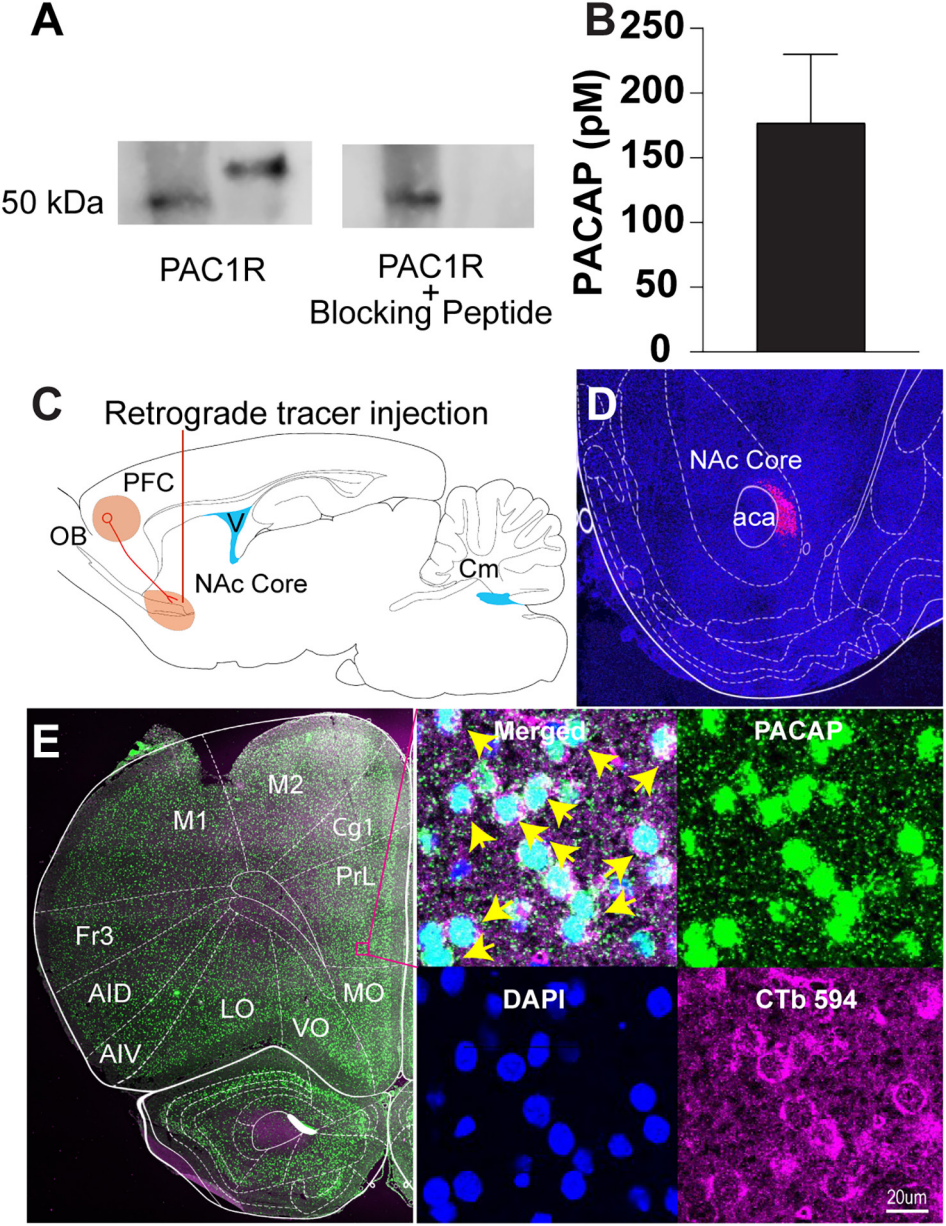
PACAP signalling in the NAcc. (A) Western blots of NAcc punch lysates confirming the presence of PACAP receptor (PAC1R), with specificity validated using a synthetic blocking peptide. (B) Quantification of endogenous PACAP levels in the NAcc extracellular lysate by microdialysis (176.70 ± 53.27 pM, mean ± SEM). (C) Diagram illustrating retrograde tracing, highlighting the neural pathway from the prefrontal cortex (PFC) to the NAcc. (D) Confocal image (10×) of the NAc injection site showing the spread of fluorescently conjugated cholera toxin subunit B (CTb 594) labelling (magenta) and cell nuclei stained with DAPI (blue), with the anterior commissure (aca) as an anatomical landmark. (E) Brain section highlighting prefrontal cortical regions with detailed anatomical labelling, including motor areas (M1 and M2), orbital cortices (MO, VO and LO) and agranular insular regions (AIV and AID). The inset (40 × composite) shows PACAP mRNA expression (green), CTb‐labelled projection neurons (magenta), nuclear DAPI staining (blue) and a merged image highlighting colocalization (yellow arrows). Abbreviations: OB (olfactory bulb), Cm (cerebellum), PrL (prelimbic cortex), Cg1 (cingulate cortex area 1), Fr3 (frontal cortex, Area 3).

We performed a series of experiments to establish the presence of the complete PACAP signalling network in the NAcc; see Figure 1. First, we confirmed that PAC1R protein expression is present in the NAcc. In Figure 1A, the left lane shows the protein ladder corresponding to 50 kDa. A protein band labelled by the PAC1R antibody is visible at 53 kDa but is absent in the presence of a PAC1R blocking peptide (Figure 1A, right image). In Figure 1B, the concentration of PACAP detected in microdialysis samples of the interstitial fluid of the rat NAcc is provided. Because PACAP can be transported across the blood–brain barrier at low concentrations [36, 37], we next confirmed its neuronal origin by assessing PACAP mRNA expression in NAcc afferents. We focused on mPFC projections to the NAcc given the importance of these inputs to motivated behaviour involving the NAcc [38]. Figure 1C illustrates the experimental procedure involving the infusion of the retrograde tracer CTb 594 into the NAcc. The localized spread of CTb 594 was confirmed to the NAcc (Figure 1D). Retrogradely labelled cells expressing PACAP mRNA were detected in the prelimbic region of the mPFC (Figure 1E). The inset images show the coexpression of CTb 594 and PACAP mRNA at a magnification of 40×. Collectively, these findings validate the existence of an endogenous central PACAP signalling network in the NAcc.

### 3.2 PACAP_1–38_ Microinjection in the NAcc Does Not Stimulate Cocaine‐Seeking Behaviour

**FIGURE 2:**
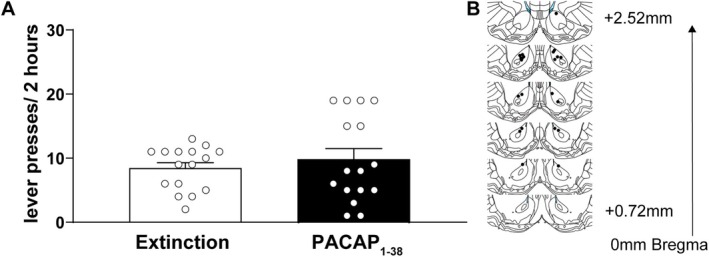
PACAP_1–38_ microinjection in the NAcc does not induce cocaine‐seeking behaviour. (A) Active lever presses (mean ± SEM) during a 2‐h session in extinction‐trained rats compared to those tested following intra‐NAcc microinjection of PACAP_1–38_ (450 ng/500 nL). Lever pressing did not significantly differ between the test sessions, indicating that PACAP_1–38_ administered into the NAcc at this dose is not sufficient to produce cocaine‐seeking behaviour (*n* = 16/condition). (B) Schematic illustration of PACAP_1–38_ microinjection sites within the NAcc, confirmed by histological analysis.

In this experiment, we examined whether microinfusion of PACAP_1–38_ is sufficient to reinstate extinguished cocaine‐seeking behaviour. The average amount of cocaine intake in this study was 1715 ± 75.60 mg/kg, and the extinction criterion was reached in an average of 10.06 ± 1.76 days. Upon reaching the extinction criterion, drug seeking was measured from each rat under an additional extinction session or during a reinstatement test in which rats received a microinfusion of PACAP_1–38_ into the NAcc. Figure 2A depicts operant responding over a 2‐h period during the final extinction session and after an intra‐NAcc infusion of PACAP_1–38_ (450 ng/500 nL; *n* = 16). A paired sample *t* test revealed no significant difference in lever pressing between the extinction and PACAP_1–38_ behavioural sessions, *t*(30) = 0.749, *p* = 0.473, Cohen's *d* = −0.257. Figure 2B depicts that the cannula placements for the rats in this study were localized to the NAcc. Collectively, our findings indicate that increased PACAP_1–38_ signalling in the NAcc does not promote drug‐seeking behaviour in rats with a history of cocaine self‐administration.

### 3.3 Intra‐NAcc PACAP_1–38_ Infusion Attenuates Cocaine‐Primed Reinstatement

**FIGURE 3:**
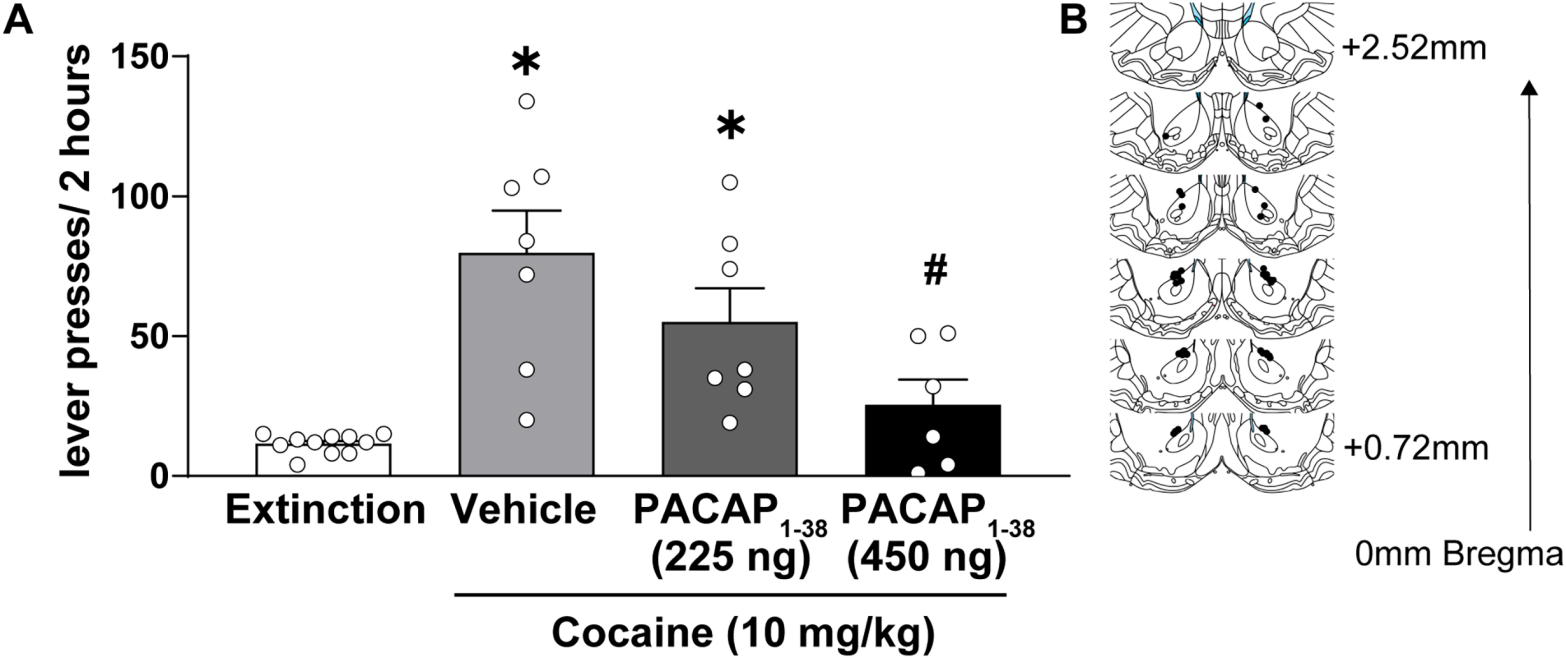
PACAP_1–38_ microinjection in the NAcc inhibits cocaine‐primed reinstatement. (A) Lever presses (mean ± SEM) during a 2‐h session by extinguished cocaine‐seeking rats reinstated with an intraperitoneal injection of 10‐mg/kg cocaine. PACAP_1–38_ microinjected into the NAcc 10 min prior to the session significantly reduced cocaine‐seeking behaviour in a dose‐dependent manner (*n* = 6–11). * and # indicate Holm–Sidak, *p* < 0.05 compared to the extinction and vehicle groups, respectively. (B) Schematic showing the injection sites within the NAcc.

To investigate this effect, rats were microinjected with vehicle or PACAP_1–38_ into the NAcc prior to a cocaine‐primed reinstatement test (Figure 3A). A one‐way ANOVA revealed a significant main effect of treatment, *F*_(3, 27)_ = 11.161, *p* = 0.00006, *ηp* ^2^ = 0.554. Post hoc pairwise comparisons revealed that cocaine (10 mg/kg) robustly reinstated drug‐seeking behaviour in rats extinguished from cocaine self‐administration, as active lever pressing in rats receiving cocaine coupled with intra‐NAcc infusions of vehicle was significantly higher than drug seeking under extinction conditions (Holm–Sidak, *p* = 0.00001, Cohen's *d* = 2.40). Cocaine‐induced reinstatement was dose‐dependently attenuated by intra‐NAcc PACAP_1–38_ infusion. Rats receiving the low dose of intra‐NAcc PACAP_1–38_ (225 ng/500 nL; *n* = 7) exhibited a significantly greater response than extinction controls (*n* = 11, Holm–Sidak, *p* = 0.00187, Cohen's *d* = 1.42) but did not differ from the vehicle + cocaine‐treated group (Holm–Sidak, *p* = 0.089, Cohen's *d* = 0.71). In contrast, administration of a high dose of intra‐NAcc PACAP_1–38_ (450 ng/500 nL; *n* = 6) significantly attenuated cocaine‐induced reinstatement behavior. Responding in this group was significantly lower than that observed in rats receiving the intra‐NAcc vehicle followed by cocaine (Holm–Sidak, *p* = 0.0009, Cohen's *d* = 1.60) and did not significantly differ from extinction controls levels (Holm–Sidak, *p* = 0.31, Cohen's *d* = 0.74).

Because reinstatement magnitude can be influenced by prior cocaine intake, we compared cumulative cocaine consumption across groups. A one‐way ANOVA revealed no significant differences in total cocaine intake, *F*_(3, 27)_ = 0.567, *p* = 0.641, *ηp* ^2^ = 0.059 (extinction: 1294 ± 76 mg/kg, vehicle: 1159 ± 66 mg/kg, 225‐ng PACAP_1–38_: 1211 ± 93 mg/kg, 450‐ng PACAP_1–38_: 1165 ± 125 mg/kg), confirming comparable cocaine exposure across conditions. Similarly, the number of extinction sessions required to meet the extinction criterion did not significantly differ among groups, *F*_(3, 27)_ = 0.214, *p* = 0.886, *ηp* ^2^ = 0.023 (extinction: 8.90 ± 0.91, vehicle: 8.43 ± 1.78, 225‐ng PACAP_1–38_: 9.71 ± 1.36, 450‐ng PACAP_1–38_: 9.83 ± 1.72), ruling out variability in extinction rate as a confounding factor.

### 3.4 PACAP_1–38_ Attenuates D1‐Like but Not D2‐Like Dopamine Receptor Agonist‐Primed Reinstatement

The NAcc integrates reward, executive function, deprivation states and other processes that regulate behaviour [3, 28, 29, 30, 31, 32]. To clarify which behavioural processes are impacted by PACAP_1–38_, we assessed its impact on drug seeking elicited by intra‐NAcc coadministration of a D1‐like or D2‐like dopamine receptor agonist, which differentially activate accumbal output pathways linked to distinct behavioural functions. Here, we assessed the effects of intra‐NAcc PACAP_1–38_ infusion coadministered with either the D1‐like dopamine receptor agonist SKF81297 or the D2‐like dopamine receptor agonist sumanirole.

**FIGURE 4:**
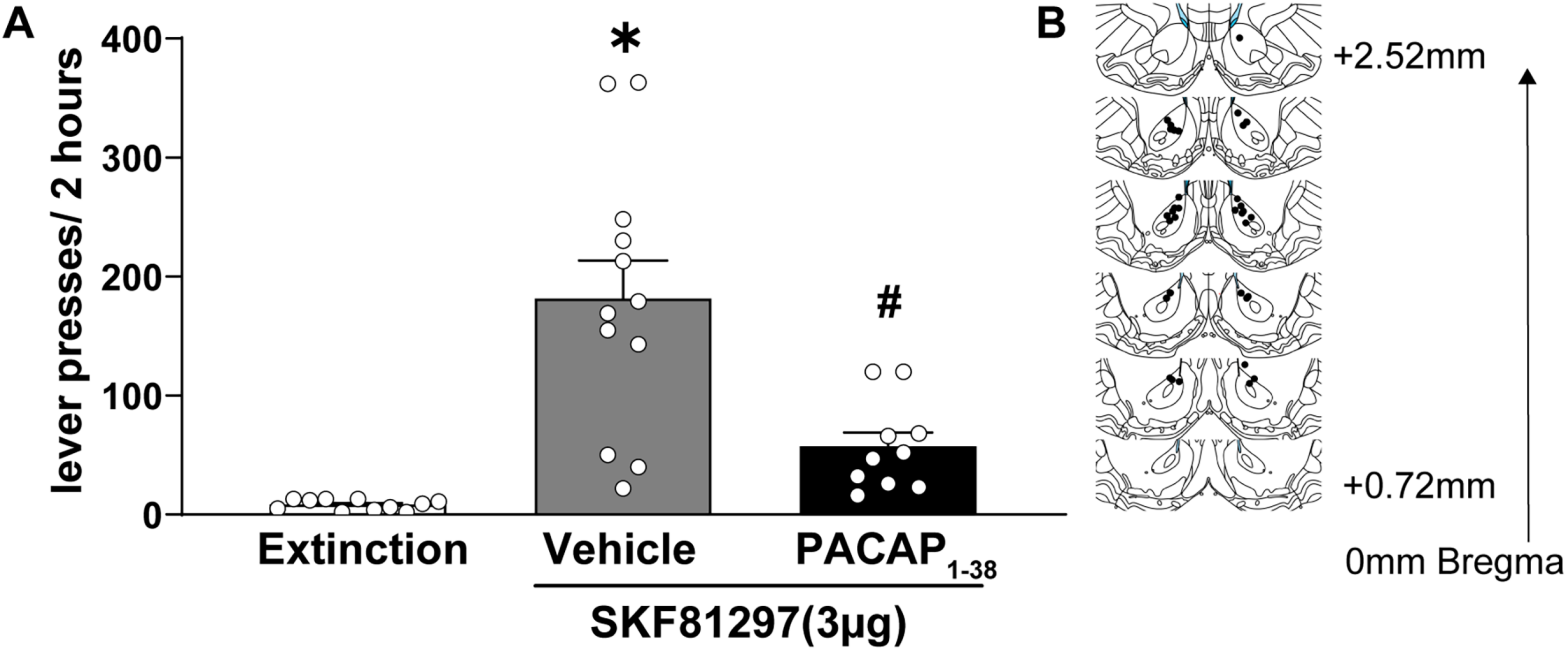
PACAP_1–38_ attenuates D1‐like dopamine receptor agonist‐primed reinstatement. (A) Lever presses (mean ± SEM) during a 2‐h session by extinguished rats following intra‐NAcc administration of the D1‐like dopamine receptor agonist SKF81297 (3 μg/500 nL). SKF81297‐induced reinstatement of cocaine‐seeking behaviour was significantly attenuated by PACAP_1–38_ (450 ng/500 nL; *n* = 10–12). * and # indicate Holm–Sidak, *p* < 0.05 compared to the extinction and vehicle groups, respectively. (B) Schematic showing the injection sites for SKF81297 and PACAP_1–38_ within the NAcc.

Figure 4A illustrates that intra‐NAcc infusion of PACAP (450 ng/500 nL) attenuated reinstatement primed by intra‐NAcc infusion of the D1‐like dopamine receptor agonist SKF81297. A one‐way ANOVA revealed a significant main effect of treatment (*F*_(2, 30)_ = 18.21, *p* = 0.000007, *ηp* ^2^ = 0.548), indicating strong PACAP‐mediated modulation of D1‐like dopamine receptor‐driven cocaine‐seeking behaviour. Post hoc pairwise comparisons using the Holm–Sidak correction confirmed that SKF81297 (3 μg/500 nL; *n* = 12) significantly increased reinstatement compared to extinction controls (*n* = 11, *p* = 0.000002, Cohen's *d* = 0.29), demonstrating that D1‐like dopamine receptor activation robustly enhances cocaine‐seeking behaviour. Coadministration of PACAP (450 ng/500 nL; *n* = 10) significantly attenuated SKF81297‐induced reinstatement (*p* = 0.0003, Cohen's *d* = 0.73). Active lever pressing in the SKF81297 + PACAP group was not significantly different from the extinction control group (*p* = 0.126, Cohen's *d* = 1.18), suggesting that PACAP effectively blocks D1‐like dopamine receptor‐mediated reinstatement.

A one‐way ANOVA revealed no significant differences in cocaine intake across rats in the various testing conditions, *F*_(2, 30)_ = 0.447, *p* = 0.644, *ηp* ^2^ = 0.029 (extinction: 1629 ± 85 mg/kg, SKF81297: 1677 ± 79 mg/kg, SKF81297 + PACAP: 1565 ± 86 mg/kg). Additionally, the number of sessions required to meet the extinction criterion did not differ among groups, *F*_(2, 30)_ = 2.441, *p* = 0.104, *ηp* ^2^ = 0.140 (extinction: 12.54 ± 2.10 sessions, SKF81297: 9.08 ± 2.42 sessions, SKF81297 + PACAP: 6.10 ± 0.98 sessions), ruling out variability in extinction learning as a confounding factor. Cannula placement for PACAP microinjections was confirmed by Cresyl violet staining, and only animals with accurate placement were used in the study (Figure 4B).

**FIGURE 5:**
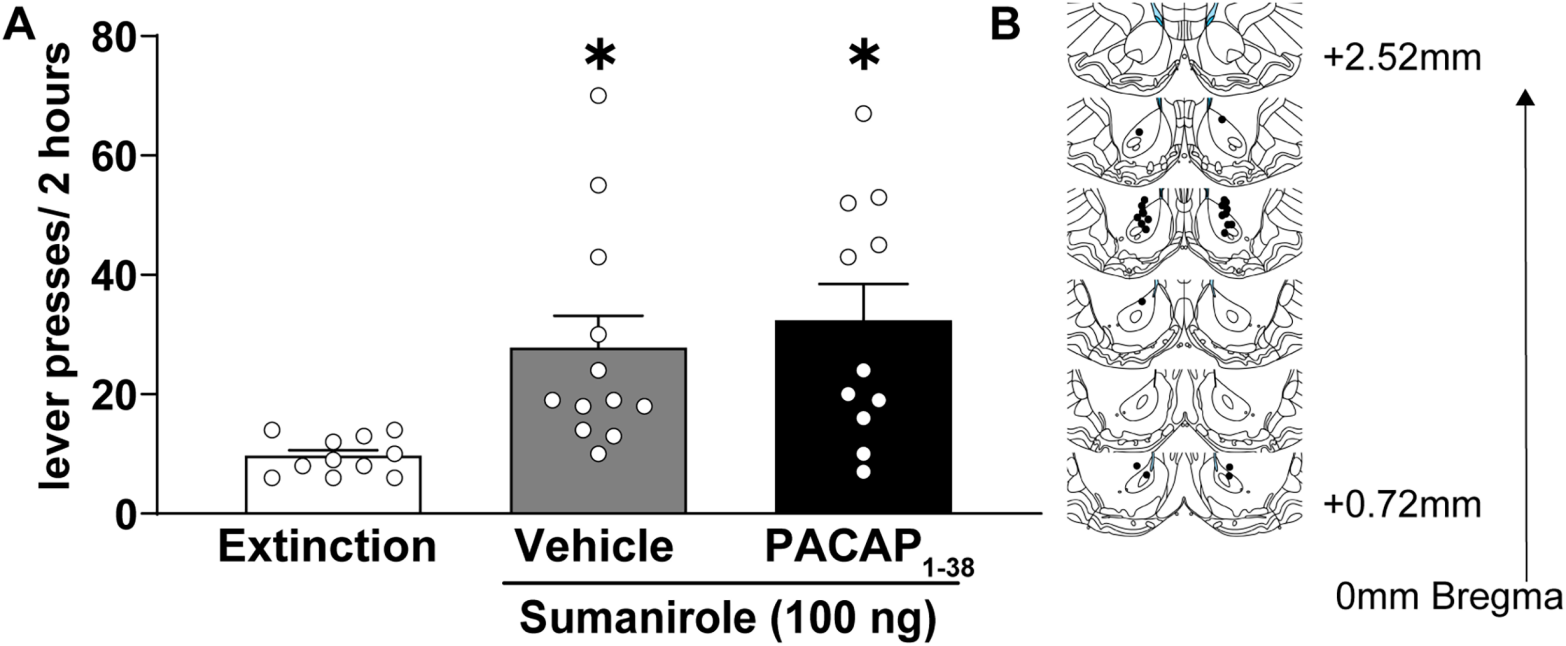
PACAP_1–38_ has no effect on D2‐like dopamine receptor agonist‐primed reinstatement in drug seeking. (A) The data (mean ± SEM) show that administration of the D2‐like dopamine receptor agonist sumanirole (100 ng/500 nL) moderately induces reinstatement in cocaine‐experienced rats and PACAP_1–38_ (450 ng/500 nL) coadministration does not inhibit it (*n* = 11–12/group). * indicates Holm–Sidak, *p* < 0.05 compared to the extinction. (B) Schematic illustrates the sumanirole and PACAP injection sites.

Next, we examined the effect of intra‐NAcc PACAP infusion on reinstatement induced by the D2‐like dopamine receptor agonist sumanirole (Figure 5A). A one‐way ANOVA revealed a significant main effect of treatment (*F*_(2, 31)_ = 6.16, *p* = 0.006, *ηp* ^2^ = 0.284), indicating that sumanirole produces a modest but significant increase in cocaine‐seeking behaviour. Post hoc pairwise comparisons using the Holm–Sidak correction confirmed that sumanirole (100 ng/500 nL; *n* = 12) significantly increased reinstatement compared to extinction controls (*n* = 11, *p* = 0.0012, Cohen's *d* = 1.35), demonstrating sumanirole facilitates drug seeking. Coadministration of PACAP (450 ng/500 nL; *n* = 11) did not significantly affect sumanirole‐induced reinstatement, as active lever pressing in the sumanirole + PACAP group remained significantly higher than extinction controls (*p* = 0.002, Cohen's *d* = 1.17) and did not differ from the sumanirole only group (*p* = 0.498, Cohen's *d* = 0.23). These findings suggest that, unlike its effect on D1‐like dopamine receptor‐mediated reinstatement, PACAP does not modulate D2‐like dopamine receptor‐driven cocaine seeking under these conditions.

A one‐way ANOVA revealed no significant differences in total cocaine intake across groups, *F*_(2, 31)_ = 0.006, *p* = 0.994, *ηp* ^2^ = 0.000 (extinction: 1710 ± 61 mg/kg, sumanirole: 1703 ± 56 mg/kg, sumanirole + PACAP: 1710 ± 61 mg/kg), confirming equivalent drug exposure across conditions. Similarly, the number of sessions required to meet the extinction criterion did not significantly differ across groups, *F*_(2, 31)_ = 1.755, *p* = 0.190, *ηp* ^2^ = 0.102 (extinction: 14.73 ± 2.28 sessions, sumanirole: 9.25 ± 2.10 sessions, sumanirole + PACAP: 14.73 ± 2.28 sessions), ruling out differences in extinction rates as a confounding variable.

## 4 Discussion

Our findings establish PACAP signalling as an integral component of the NAcc that selectively regulates drug‐seeking behaviour through dopamine receptor‐specific mechanisms. Supporting this, we demonstrate that PACAP and its cognate receptor, PAC1R, are endogenously expressed in rat NAcc. Using retrograde tracing and in situ hybridization, we reveal the presence of PACAP mRNA‐expressing neurons projecting from the mPFC to the NAcc. In the cocaine self‐administration and reinstatement paradigm, PACAP_1–38_ infusions into the NAcc did not reinstate cocaine seeking in extinguished rats but blocked cocaine‐primed reinstatement. Further, PACAP_1–38_ specifically inhibited reinstatement driven by intra‐NAcc coinfusion of the D1‐like dopamine receptor agonist SKF81297, without altering behaviour produced by intra‐NAcc coinfusion of the D2‐like dopamine receptor agonist sumanirole. These results establish PACAP_1–38_ as an endogenous neuromodulator within the NAcc, selectively regulating motivated behaviour by targeting specific dopaminergic pathways. By demonstrating PACAP_1–38_'s ability to suppress cocaine seeking through D1‐like dopamine receptor‐dependent mechanisms and its localization in corticostriatal projections, our study highlights its potential role in CUD, a disorder characterized by dysregulated reward, emotional and cognitive processes [1, 2, 4, 5, 6, 7, 8, 9].

Our first objective was to investigate the potential existence of a PACAP_1–38_ signalling network in the rat NAcc. Western blot analysis confirmed the NAcc expression of PAC1R protein, the primary effector of PACAP_1–38_ signalling [39]. This is the first demonstration of PAC1R protein in the NAcc of Sprague–Dawley rats, which extends existing observations documenting the presence of PAC1R protein in the NAcc of Wistar rats [40, 41]. In vivo peptide microdialysis samples of NAcc interstitial fluid contained the PACAP peptide, which is the first direct evidence of extracellular PACAP in this region. Lastly, PACAP mRNA was detected in NAcc‐projecting afferents from the mPFC, indicating that PACAP may be coreleased with glutamate at corticostriatal synapses. This is an important finding because it suggests that neuronal release is a possible source of the extracellular PACAP detected in the NAcc. While the prefrontal cortex appears to be one source, other PACAP‐expressing regions, including the paraventricular thalamus, hypothalamus, bed nucleus of the stria terminalis (BNST) and amygdala, also send dense projections to the NAcc [20, 42, 43, 44]. In addition, PACAP can be transported across blood–brain barriers [45]. Future research is needed to examine the functional relevance of the various sources of PACAP to NAcc function. Collectively, these findings indicate the existence of a PACAP signalling network within the NAcc.

To assess its functional relevance, we next investigated the impact of PACAP signalling in the NAcc on cocaine‐seeking behaviour. We found that intra‐NAcc infusion of PACAP alone did not significantly modify conditions. However, intra‐NAcc infusion of PACAP blocked reinstatement primed by a cocaine injection (10 mg/kg, ip). These results suggest that PACAP_1–38_ signalling in the NAcc suppresses motivated behaviour, which aligns with prior work showing that PACAP_1–38_ signalling in this region reduces hedonic drive [46, 47]. These findings are also consistent with prior observations that PACAP_1–38_ signalling increases the activity of astrocytic glutamatergic mechanisms, including GLT‐1 [48] and system xc [49], both of which suppress cocaine seeking by depressing excitatory signalling [50, 51, 52, 53, 54]. An additional relevant finding is that PACAP_1–38_‐induced suppression of palatable food when infused into the NAcc mimics the actions of increased GABA signalling in the NAcc [47]. Collectively, these findings indicate that PACAP_1–38_ signalling mimics or enhances inhibitory signals in the NAcc that suppress motivated behaviour.

Our finding that PACAP suppresses drug‐seeking behaviour contrasts with several prior studies, which likely reflect the complexity of PACAP signalling in the brain. Infusion of PACAP_1–38_ into the BNST has been shown to reinstate, rather than inhibit, cocaine‐ and stress‐induced reinstatement of drug‐seeking behaviour [55], suggesting region‐specific effects of PACAP signalling. Consistent with region‐specific effects on behaviour, PACAP has been reported to produce either excitatory or inhibitory effects depending on the brain area studied [47, 56, 57, 58, 59]. In addition to regional variation, strain‐specific factors may contribute to opposing behavioural outcomes. For instance, the inhibition of PACAP signalling in the nucleus accumbens shell or core increased alcohol‐seeking behaviour in alcohol‐preferring Scr:sP rats but had no effect in outbred Wistar rats [40, 41]. The differences between these and our study may also arise from the manipulation used to alter PACAP signalling. While we activated PACAP receptors using PACAP_1–38_, the aforementioned alcohol studies used PACAP_6–38_ or viral‐mediated receptor knockdown to inhibit PACAP receptor activation [40, 41], which would block activation by PACAP_1–38_ as well as by PACAP_1–27_ or other endogenous agonists of PACAP receptors, including vasoactive intestinal peptide [60, 61]. Isoform‐specific effects have also been reported. In the NAcc, PACAP_1–38_ and but not PACAP_1–27_ reduced alcohol consumption, whereas in the shell, the reverse pattern was reported [15]. These findings highlight the importance of brain region, receptor isoform, strain and pharmacological specificity in interpreting the behavioural effects of PACAP signalling.

In addition, PACAP's behavioural effects may depend on the molecular context in which it is engaged. Specifically, we observed behavioural suppression in the presence of cocaine but not in its absence. One possible explanation is that the behaviour‐suppressing effects associated with PACAP receptor activation require molecular events triggered by cocaine exposure, such as rapid activation of dopamine receptors. If so, this possibility could also account for discrepancies observed between cocaine and other stimuli, such as stress or alcohol. Cocaine induces a rapid, phasic increase in dopamine levels within the nucleus accumbens by inhibiting dopamine reuptake via the dopamine transporter [62]. In contrast, alcohol elicits a lower magnitude elevation in accumbal dopamine, primarily through its action in the ventral tegmental area [63, 64]. Furthermore, chronic exposure to cocaine alters the expression of both D1‐like and D2‐like dopamine receptors in the nucleus accumbens, which may further contribute to divergent behavioural outcomes [65, 66]. Given this possibility, we next tested whether PACAP alters reinstatement produced by direct stimulation of dopamine receptors in the NAcc.

Coinfusion of PACAP_1–38_ attenuated the robust reinstatement produced by intra‐NAcc administration of the D1‐like dopamine receptor agonist SKF81297. In contrast, PACAP_1–38_ infusions did not significantly alter the modest reinstatement produced by intra‐NAcc infusion of the D2‐like dopamine receptor agonist sumanirole. The selective inhibition of reinstatement induced by D1‐like but not D2‐like dopamine receptor agonism in the NAcc may offer insight into the specific behavioural mechanisms influenced by PACAP_1–38_. Consistent with this, activation of D1‐like and D2‐like dopamine receptors in the NAcc is known to recruit distinct neural circuits and motivational processes [67]. For example, chemogenetic activation of NAcc D1‐expressing medium spiny neurons (MSNs) increases effort for a palatable reward while decreasing overall food consumption, whereas the activation of D2‐expressing MSNs increases immediate food intake while reducing effortful reward seeking and locomotion [68, 69]. D1‐ and D2‐like dopamine receptor signalling within the NAcc and other striatal subregions also has distinct functions involving cognitive and reward processing [70, 71, 72].

Collectively, the present and past findings are advancing our understanding of central PACAP signalling and underscore the importance of continued research to define its behavioural and mechanistic roles to determine whether this system can support the development of targeted therapeutics. The complexity of PACAP's behavioural effects, including its ability to both enhance and suppress motivated behaviour, may limit the therapeutic utility of PACAP receptor‐targeting compounds that are broadly distributed throughout the brain. Realizing the therapeutic potential of PACAP signalling may require future strategies capable of selectively targeting the subset of PACAP's actions that result in suppressed behaviours.

One promising direction is to determine whether peripheral PACAP signalling, or that of related gut‐derived hormones, can be used to selectively activate central PACAP signalling mechanisms that modulate behavioural suppression. A similar approach has proven effective with GLP‐1, a related peptide in the secretin superfamily that includes PACAP [13, 73]. Both PACAP and GLP‐1 function through signalling systems that span peripheral and central domains, and both are thought to have evolved to regulate feeding and other complex motivated behaviours [13, 73]. GLP‐1 receptor agonists restricted to peripheral tissues can regulate specific brain circuits [74], suggesting that a similar degree of precise targeting may be achievable with PACAP‐based therapies. Although PACAP and GLP‐1 share structural and functional features, they may regulate different aspects of behaviour due to partially distinct receptor distributions and unique pharmacodynamic properties [20, 75, 76, 77, 78, 79, 80, 81, 82]. While both receptor systems are coexpressed in brain regions involved in affect and motivation, PACAP receptors are expressed in areas such as the cerebellum and specific cortical regions, where GLP‐1 receptor expression is minimal or absent [20, 75, 76, 78, 79]. Even when receptors are coexpressed within the same brain region, they may elicit distinct pharmacodynamic responses due to differences in receptor signalling, localization or downstream effectors. Hence, PACAP, GLP‐1 and potentially other members of the secretin peptide family may exert synergistic, complementary or distinct effects on central processes that regulate behaviour. Our findings underscore the need to more precisely characterize the behavioural functions of PACAP alone or in combination with related peptides.

## Author Contributions

D.A.B. conceptualized and designed the studies. S.B., G.S., E.M.H., L.K., B.M. and N.J.R. collected the data. D.A.B., S.C., E.M.H., L.K., G.S. and S.B. analysed the data and wrote the manuscript. All authors read and approved the manuscript.

## Ethics Statement

The experimental protocol involving animals was ethically reviewed and approved in accordance with guidelines for the care and use of laboratory animals, MU IACUC (AR‐3815).

## Conflicts of Interest

D.A.B. is the co‐founder of, and owns shares in, Promentis Pharmaceuticals. Promentis is developing glutamatergic compounds to treat impulse control disorders but was not involved in these studies.

## Data Availability

The data that support the findings of this study are available from the corresponding author upon reasonable request.

## References

[1] R. Peters , N. Ee , J. Peters , et al., “Common Risk Factors for Major Noncommunicable Disease, a Systematic Overview of Reviews and Commentary: The Implied Potential for Targeted Risk Reduction,” Therapeutic Advances in Chronic Disease 10 (2019): 2040622319880392, 10.1177/2040622319880392. 31662837 PMC6794648

[2] R. Uddin , E. Y. Lee , S. R. Khan , M. S. Tremblay , and A. Khan , “Clustering of Lifestyle Risk Factors for Non‐Communicable Diseases in 304,779 Adolescents From 89 Countries: A Global Perspective,” Preventive Medicine 131 (2020): 105955, 10.1016/j.ypmed.2019.105955. 31862205

[3] G. J. Mogenson , D. J. Jones , and C. Y. Yim , “From Motivation to Action: Functional Interface Between the Limbic System and the Motor System,” Progress in Neurobiology 14 (1980): 69–97. 6999537 10.1016/0301-0082(80)90018-0

[4] A. Verdejo‐Garcia , L. Clark , J. Verdejo‐Roman , et al., “Neural Substrates of Cognitive Flexibility in Cocaine and Gambling Addictions,” British Journal of Psychiatry 207 (2015): 158–164, 10.1192/bjp.bp.114.152223. 26045346

[5] F. Lange , C. Seer , and B. Kopp , “Cognitive Flexibility in Neurological Disorders: Cognitive Components and Event‐Related Potentials,” Neuroscience and Biobehavioral Reviews 83 (2017): 496–507, 10.1016/j.neubiorev.2017.09.011. 28903059

[6] T. Ramey and P. S. Regier , “Cognitive Impairment in Substance Use Disorders,” CNS Spectrums 24 (2019): 102–113, 10.1017/S1092852918001426. 30591083 PMC6599555

[7] L. A. Keyser‐Marcus , T. Ramey , J. Bjork , A. Adams , and F. G. Moeller , “Development and Feasibility Study of an Addiction‐Focused Phenotyping Assessment Battery,” American Journal on Addictions 30 (2021): 398–405, 10.1111/ajad.13170. 33908104 PMC8243823

[8] A. L. Watts , R. D. Latzman , C. L. Boness , et al., “New Approaches to Deep Phenotyping in Addictions,” Psychology of Addictive Behaviors 37 (2023): 361–375, 10.1037/adb0000878. 36174150 PMC10050231

[9] K. J. Ressler and H. S. Mayberg , “Targeting Abnormal Neural Circuits in Mood and Anxiety Disorders: From the Laboratory to the Clinic,” Nature Neuroscience 10 (2007): 1116–1124, 10.1038/nn1944. 17726478 PMC2444035

[10] V. K. Gribkoff and L. K. Kaczmarek , “The Need for New Approaches in CNS Drug Discovery: Why Drugs Have Failed, and What Can Be Done to Improve Outcomes,” Neuropharmacology 120 (2017): 11–19, 10.1016/j.neuropharm.2016.03.021. 26979921 PMC5820030

[11] H. Geerts , J. Wikswo , P. H. van der Graaf , et al., “Quantitative Systems Pharmacology for Neuroscience Drug Discovery and Development: Current Status, Opportunities, and Challenges,” CPT: Pharmacometrics & Systems Pharmacology 9 (2020): 5–20, 10.1002/psp4.12478. 31674729 PMC6966183

[12] J. Y. Hansen , G. Shafiei , R. D. Markello , et al., “Mapping Neurotransmitter Systems to the Structural and Functional Organization of the Human Neocortex,” Nature Neuroscience 25 (2022): 1569–1581, 10.1038/s41593-022-01186-3. 36303070 PMC9630096

[13] R. Sekar , L. Wang , and B. K. Chow , “Central Control of Feeding Behavior by the Secretin, PACAP, and Glucagon Family of Peptides,” Frontiers in Endocrinology 8 (2017): 18, 10.3389/fendo.2017.00018. 28223965 PMC5293785

[14] K. Sureshkumar , A. Saenz , S. M. Ahmad , and K. Lutfy , “The PACAP/PAC1 Receptor System and Feeding,” Brain Sciences 12, no. 1 (2021): 13, 10.3390/brainsci12010013. 35053757 PMC8773599

[15] A. T. Gargiulo , B. E. Pirino , G. R. Curtis , and J. R. Barson , “Effects of Pituitary Adenylate Cyclase‐Activating Polypeptide Isoforms in Nucleus Accumbens Subregions on Ethanol Drinking,” Addiction Biology 26 (2021): e12972, 10.1111/adb.12972. 33020973 PMC8019681

[16] F. Sundler , E. Ekblad , A. Absood , R. Hakanson , K. Koves , and A. Arimura , “Pituitary Adenylate Cyclase Activating Peptide: A Novel Vasoactive Intestinal Peptide‐Like Neuropeptide in the Gut,” Neuroscience 46 (1992): 439–454, 10.1016/0306-4522(92)90064-9. 1542417

[17] J. P. Vu , D. Goyal , L. Luong , et al., “PACAP Intraperitoneal Treatment Suppresses Appetite and Food Intake via PAC1 Receptor in Mice by Inhibiting Ghrelin and Increasing GLP‐1 and Leptin,” American Journal of Physiology. Gastrointestinal and Liver Physiology 309 (2015): G816–G825, 10.1152/ajpgi.00190.2015. 26336928 PMC4652141

[18] M. L. Lehmann , T. Mustafa , A. M. Eiden , M. Herkenham , and L. E. Eiden , “PACAP‐Deficient Mice Show Attenuated Corticosterone Secretion and Fail to Develop Depressive Behavior During Chronic Social Defeat Stress,” Psychoneuroendocrinology 38 (2013): 702–715, 10.1016/j.psyneuen.2012.09.006. 23062748 PMC3652373

[19] H. L. Zhang , Y. Sun , Z. J. Wu , et al., “Hippocampal PACAP Signaling Activation Triggers a Rapid Antidepressant Response,” Military Medical Research 11 (2024): 49, 10.1186/s40779-024-00548-1. 39044298 PMC11265467

[20] L. Zhang , V. S. Hernandez , C. R. Gerfen , et al., “Behavioral Role of PACAP Signaling Reflects Its Selective Distribution in Glutamatergic and GABAergic Neuronal Subpopulations,” eLife 10 (2021): e61718, 10.7554/eLife.61718. 33463524 PMC7875564

[21] D. Chen , G. F. Buchanan , J. M. Ding , J. Hannibal , and M. U. Gillette , “Pituitary Adenylyl Cyclase‐Activating Peptide: A Pivotal Modulator of Glutamatergic Regulation of the Suprachiasmatic Circadian Clock,” Proceedings of the National Academy of Sciences of the United States of America 96 (1999): 13468–13473, 10.1073/pnas.96.23.13468. 10557344 PMC23971

[22] K. J. Clancy , Q. Devignes , P. Kumar , et al., “Circulating PACAP Levels Are Associated With Increased Amygdala‐Default Mode Network Resting‐State Connectivity in Posttraumatic Stress Disorder,” Neuropsychopharmacology 48 (2023): 1245–1254, 10.1038/s41386-023-01593-5. 37161077 PMC10267202

[23] H. Hashimoto , N. Shintani , M. Tanida , A. Hayata , R. Hashimoto , and A. Baba , “PACAP Is Implicated in the Stress Axes,” Current Pharmaceutical Design 17 (2011): 985–989, 10.2174/138161211795589382. 21524255 PMC3179129

[24] K. J. Ressler , K. B. Mercer , B. Bradley , et al., “Post‐Traumatic Stress Disorder Is Associated With PACAP and the PAC1 Receptor,” Nature 470 (2011): 492–497, 10.1038/nature09856. 21350482 PMC3046811

[25] A. T. Gargiulo , G. R. Curtis , and J. R. Barson , “Pleiotropic Pituitary Adenylate Cyclase‐Activating Polypeptide (PACAP): Novel Insights Into the Role of PACAP in Eating and Drug Intake,” Brain Research 1729 (2020): 146626, 10.1016/j.brainres.2019.146626. 31883848 PMC6953419

[26] A. Stojakovic , S. M. Ahmad , S. Malhotra , Z. Afzal , M. Ahmed , and K. Lutfy , “The Role of Pituitary Adenylyl Cyclase‐Activating Polypeptide in the Motivational Effects of Addictive Drugs,” Neuropharmacology 171 (2020): 108109, 10.1016/j.neuropharm.2020.108109. 32325064

[27] C. E. Van Doorn , M. M. Zelows , and A. A. Jaramillo , “Pituitary Adenylate Cyclase‐Activating Polypeptide Plays a Role in Neuropsychiatric and Substance Use Disorders: Sex‐Specific Perspective,” Frontiers in Neuroscience 19 (2025): 1545810, 10.3389/fnins.2025.1545810. 39975969 PMC11835941

[28] S. B. Floresco , “The Nucleus Accumbens: An Interface Between Cognition, Emotion, and Action,” Annual Review of Psychology 66 (2015): 25–52, 10.1146/annurev-psych-010213-115159. 25251489

[29] M. D. Scofield , J. A. Heinsbroek , C. D. Gipson , et al., “The Nucleus Accumbens: Mechanisms of Addiction Across Drug Classes Reflect the Importance of Glutamate Homeostasis,” Pharmacological Reviews 68 (2016): 816–871, 10.1124/pr.116.012484. 27363441 PMC4931870

[30] Y. Goto and A. A. Grace , “Limbic and Cortical Information Processing in the Nucleus Accumbens,” Trends in Neurosciences 31 (2008): 552–558, 10.1016/j.tins.2008.08.002. 18786735 PMC2884964

[31] G. J. Mogenson , “Limbic‐Motor Integration,” in Progress in Psychobiology and Physiological Psychology, eds. A. N. Epstein and A. R. Morrison (Academic Press, 1987): 117–170.

[32] S. J. McDougall , Z. Y. Ong , R. Heller , et al., “Viscerosensory Signalling to the Nucleus Accumbens via the Solitary Tract Nucleus,” Journal of Neurochemistry 168 (2024): 3116–3131, 10.1111/jnc.16180. 39032068

[33] E. M. Hess , S. N. Kassel , G. Simandl , et al., “Genetic Disruption of System xc‐Mediated Glutamate Release From Astrocytes Increases Negative‐Outcome Behaviors While Preserving Basic Brain Function in Rat,” Journal of Neuroscience 43 (2023): 2349–2361, 10.1523/JNEUROSCI.1525-22.2023. 36788029 PMC10072291

[34] A. Madayag , K. S. Kau , D. Lobner , J. R. Mantsch , S. Wisniewski , and D. A. Baker , “Drug‐Induced Plasticity Contributing to Heightened Relapse Susceptibility: Neurochemical Changes and Augmented Reinstatement in High‐Intake Rats,” Journal of Neuroscience 30 (2010): 210–217, 10.1523/JNEUROSCI.1342-09.2010. 20053903 PMC2823262

[35] G. Paxinos and C. Watson , The Rat Brain in Stereotaxic Coordinates: Hard Cover Edition (Academic Press, 2006).

[36] N. Nonaka , W. A. Banks , H. Mizushima , S. Shioda , and J. E. Morley , “Regional Differences in PACAP Transport Across the Blood‐Brain Barrier in Mice: A Possible Influence of Strain, Amyloid Beta Protein, and Age,” Peptides 23 (2002): 2197–2202, 10.1016/s0196-9781(02)00248-6. 12535699

[37] W. A. Banks , A. J. Kastin , G. Komaki , and A. Arimura , “Passage of Pituitary Adenylate Cyclase Activating Polypeptide1‐27 and Pituitary Adenylate Cyclase Activating Polypeptide1‐38 Across the Blood‐Brain Barrier,” Journal of Pharmacology and Experimental Therapeutics 267 (1993): 690–696. 8246142

[38] K. McFarland and P. W. Kalivas , “The Circuitry Mediating Cocaine‐Induced Reinstatement of Drug‐Seeking Behavior,” Journal of Neuroscience 21 (2001): 8655–8663, 10.1523/JNEUROSCI.21-21-08655.2001. 11606653 PMC6762812

[39] M. N. Boucher , V. May , K. M. Braas , and S. E. Hammack , “PACAP Orchestration of Stress‐Related Responses in Neural Circuits,” Peptides 142 (2021): 170554, 10.1016/j.peptides.2021.170554. 33865930 PMC8592028

[40] M. A. Minnig , A. Blasio , A. Ferragud , et al., “Pituitary Adenylate Cyclase‐Activating Polypeptide Type 1 Receptor Within the Nucleus Accumbens Core Mediates Excessive Alcohol Drinking in Alcohol‐Preferring Rats,” Neuropharmacology 212 (2022): 109063, 10.1016/j.neuropharm.2022.109063. 35460713 PMC10342914

[41] M. A. Minnig , T. Park , M. Echeveste Sanchez , P. Cottone , and V. Sabino , “Viral‐Mediated Knockdown of Nucleus Accumbens Shell PAC1 Receptor Promotes Excessive Alcohol Drinking in Alcohol‐Preferring Rats,” Frontiers in Behavioral Neuroscience 15 (2021): 787362, 10.3389/fnbeh.2021.787362. 34924973 PMC8678417

[42] G. R. Curtis , K. Oakes , and J. R. Barson , “Expression and Distribution of Neuropeptide‐Expressing Cells Throughout the Rodent Paraventricular Nucleus of the Thalamus,” Frontiers in Behavioral Neuroscience 14 (2020): 634163, 10.3389/fnbeh.2020.634163. 33584216 PMC7873951

[43] X. Dong , S. Li , and G. J. Kirouac , “Collateralization of Projections From the Paraventricular Nucleus of the Thalamus to the Nucleus Accumbens, Bed Nucleus of the Stria Terminalis, and Central Nucleus of the Amygdala,” Brain Structure & Function 222 (2017): 3927–3943, 10.1007/s00429-017-1445-8. 28528379

[44] J. Hannibal , “Pituitary Adenylate Cyclase‐Activating Peptide in the Rat Central Nervous System: An Immunohistochemical and In Situ Hybridization Study,” Journal of Comparative Neurology 453 (2002): 389–417, 10.1002/cne.10418. 12389210

[45] A. Somogyvari‐Vigh , W. Pan , D. Reglodi , A. J. Kastin , and A. Arimura , “Effect of Middle Cerebral Artery Occlusion on the Passage of Pituitary Adenylate Cyclase Activating Polypeptide Across the Blood‐Brain Barrier in the Rat,” Regulatory Peptides 91 (2000): 89–95, 10.1016/s0167-0115(00)00123-3. 10967205

[46] M. M. Hurley , M. R. Robble , G. Callan , S. Choi , and R. A. Wheeler , “Pituitary Adenylate Cyclase‐Activating Polypeptide (PACAP) Acts in the Nucleus Accumbens to Reduce Hedonic Drive,” International Journal of Obesity 43 (2019): 928–932, 10.1038/s41366-018-0154-6. 30082747 PMC6363914

[47] M. M. Hurley , B. Maunze , M. E. Block , et al., “Pituitary Adenylate‐Cyclase Activating Polypeptide Regulates Hunger‐ and Palatability‐Induced Binge Eating,” Frontiers in Neuroscience 10 (2016): 383, 10.3389/fnins.2016.00383. 27597817 PMC4993128

[48] M. Figiel and J. Engele , “Pituitary Adenylate Cyclase‐Activating Polypeptide (PACAP), a Neuron‐Derived Peptide Regulating Glial Glutamate Transport and Metabolism,” Journal of Neuroscience 20 (2000): 3596–3605. 10804201 10.1523/JNEUROSCI.20-10-03596.2000 PMC6772687

[49] L. Kong , R. Albano , A. Madayag , et al., “Pituitary Adenylate Cyclase‐Activating Polypeptide Orchestrates Neuronal Regulation of the Astrocytic Glutamate‐Releasing Mechanism System x_c_ ^−^ ,” Journal of Neurochemistry 137 (2016): 384–393, 10.1111/jnc.13566. 26851652 PMC4878137

[50] K. S. Kau , A. Madayag , J. R. Mantsch , M. D. Grier , O. Abdulhameed , and D. A. Baker , “Blunted Cystine‐Glutamate Antiporter Function in the Nucleus Accumbens Promotes Cocaine‐Induced Drug Seeking,” Neuroscience 155 (2008): 530–537, 10.1016/j.neuroscience.2008.06.010. 18601982 PMC2614296

[51] D. A. Baker , K. McFarland , R. W. Lake , et al., “Neuroadaptations in Cystine‐Glutamate Exchange Underlie Cocaine Relapse,” Nature Neuroscience 6 (2003): 743–749, 10.1038/nn1069. 12778052

[52] L. A. Knackstedt , R. I. Melendez , and P. W. Kalivas , “Ceftriaxone Restores Glutamate Homeostasis and Prevents Relapse to Cocaine Seeking,” Biological Psychiatry 67 (2010): 81–84. 19717140 10.1016/j.biopsych.2009.07.018 PMC2795043

[53] M. M. Moran , K. McFarland , R. I. Melendez , P. W. Kalivas , and J. K. Seamans , “Cystine/Glutamate Exchange Regulates Metabotropic Glutamate Receptor Presynaptic Inhibition of Excitatory Transmission and Vulnerability to Cocaine Seeking,” Journal of Neuroscience 25 (2005): 6389–6393. 16000629 10.1523/JNEUROSCI.1007-05.2005 PMC1413952

[54] H. Trantham‐Davidson , R. T. LaLumiere , K. J. Reissner , P. W. Kalivas , and L. A. Knackstedt , “Ceftriaxone Normalizes Nucleus Accumbens Synaptic Transmission, Glutamate Transport, and Export Following Cocaine Self‐Administration and Extinction Training,” Journal of Neuroscience 32 (2012): 12406–12410, 10.1523/JNEUROSCI.1976-12.2012. 22956831 PMC3465971

[55] O. W. Miles , E. A. Thrailkill , A. K. Linden , V. May , M. E. Bouton , and S. E. Hammack , “Pituitary Adenylate Cyclase‐Activating Peptide in the Bed Nucleus of the Stria Terminalis Mediates Stress‐Induced Reinstatement of Cocaine Seeking in Rats,” Neuropsychopharmacology 43 (2018): 978–986, 10.1038/npp.2017.135. 28656976 PMC5854788

[56] S. Ortega‐Tinoco , M. Padilla‐Orozco , F. Hernandez‐Vazquez , et al., “PACAP Induces Increased Excitability in D1‐ and D2‐Expressing Nucleus Accumbens Medium Spiny Neurons,” Brain Research Bulletin 224 (2025): 111323, 10.1016/j.brainresbull.2025.111323. 40147707

[57] G. C. Johnson , R. Parsons , V. May , and S. E. Hammack , “The Role of Pituitary Adenylate Cyclase‐Activating Polypeptide (PACAP) Signaling in the Hippocampal Dentate Gyrus,” Frontiers in Cellular Neuroscience 14 (2020): 111, 10.3389/fncel.2020.00111. 32425759 PMC7203336

[58] R. D. Taylor , M. G. Madsen , M. Krause , M. Sampedro‐Castaneda , M. Stocker , and P. Pedarzani , “Pituitary Adenylate Cyclase‐Activating Polypeptide (PACAP) Inhibits the Slow Afterhyperpolarizing Current sIAHP in CA1 Pyramidal Neurons by Activating Multiple Signaling Pathways,” Hippocampus 24 (2014): 32–43, 10.1002/hipo.22201. 23996525 PMC3920641

[59] J. Ster , F. de Bock , F. Bertaso , et al., “Epac Mediates PACAP‐Dependent Long‐Term Depression in the Hippocampus,” Journal of Physiology 587 (2009): 101–113, 10.1113/jphysiol.2008.157461. 19001039 PMC2670026

[60] T. Hirabayashi , T. Nakamachi , and S. Shioda , “Discovery of PACAP and Its Receptors in the Brain,” Journal of Headache and Pain 19 (2018): 28, 10.1186/s10194-018-0855-1. 29619773 PMC5884755

[61] D. Vaudry , A. Falluel‐Morel , S. Bourgault , et al., “Pituitary Adenylate Cyclase‐Activating Polypeptide and Its Receptors: 20 Years After the Discovery,” Pharmacological Reviews 61 (2009): 283–357, 10.1124/pr.109.001370. 19805477

[62] E. B. Oleson , S. Talluri , S. R. Childers , et al., “Dopamine Uptake Changes Associated With Cocaine Self‐Administration,” Neuropsychopharmacology 34 (2009): 1174–1184, 10.1038/npp.2008.186. 18923398 PMC2656581

[63] H. Morikawa and R. A. Morrisett , “Ethanol Action on Dopaminergic Neurons in the Ventral Tegmental Area: Interaction With Intrinsic Ion Channels and Neurotransmitter Inputs,” International Review of Neurobiology 91 (2010): 235–288, 10.1016/S0074-7742(10)91008-8. 20813245 PMC2936723

[64] C. You , B. Vandegrift , and M. S. Brodie , “Ethanol Actions on the Ventral Tegmental Area: Novel Potential Targets on Reward Pathway Neurons,” Psychopharmacology 235 (2018): 1711–1726, 10.1007/s00213-018-4875-y. 29549390 PMC5949141

[65] K. Park , N. D. Volkow , Y. Pan , and C. Du , “Chronic Cocaine Dampens Dopamine Signaling During Cocaine Intoxication and Unbalances D1 Over D2 Receptor Signaling,” Journal of Neuroscience 33 (2013): 15827–15836, 10.1523/JNEUROSCI.1935-13.2013. 24089490 PMC3787501

[66] K. Feltmann , D. O. Borroto‐Escuela , J. Ruegg , et al., “Effects of Long‐Term Alcohol Drinking on the Dopamine D2 Receptor: Gene Expression and Heteroreceptor Complexes in the Striatum in Rats,” Alcoholism, Clinical and Experimental Research 42 (2018): 338–351, 10.1111/acer.13568. 29205397 PMC5817245

[67] A. M. Klawonn and R. C. Malenka , “Nucleus Accumbens Modulation in Reward and Aversion,” Cold Spring Harbor Symposia on Quantitative Biology 83 (2018): 119–129, 10.1101/sqb.2018.83.037457. 30674650 PMC6650377

[68] S. L. Cole , M. J. F. Robinson , and K. C. Berridge , “Optogenetic Self‐Stimulation in the Nucleus Accumbens: D1 Reward Versus D2 Ambivalence,” PLoS ONE 13 (2018): e0207694, 10.1371/journal.pone.0207694. 30496206 PMC6264872

[69] R. Walle , A. Petitbon , G. R. Fois , et al., “Nucleus Accumbens D1‐ and D2‐Expressing Neurons Control the Balance Between Feeding and Activity‐Mediated Energy Expenditure,” Nature Communications 15 (2024): 2543, 10.1038/s41467-024-46874-9. PMC10958053 38514654

[70] J. Sala‐Bayo , L. Fiddian , S. R. O. Nilsson , et al., “Dorsal and Ventral Striatal Dopamine D1 and D2 Receptors Differentially Modulate Distinct Phases of Serial Visual Reversal Learning,” Neuropsychopharmacology 45 (2020): 736–744, 10.1038/s41386-020-0612-4. 31940660 PMC7075980

[71] T. Macpherson , J. Y. Kim , and T. Hikida , “Nucleus Accumbens Core Dopamine D2 Receptor‐Expressing Neurons Control Reversal Learning but Not Set‐Shifting in Behavioral Flexibility in Male Mice,” Frontiers in Neuroscience 16 (2022): 885380, 10.3389/fnins.2022.885380. 35837123 PMC9275008

[72] J. E. Zachry , M. G. Kutlu , H. J. Yoon , et al., “D1 and D2 Medium Spiny Neurons in the Nucleus Accumbens Core Have Distinct and Valence‐Independent Roles in Learning,” Neuron 112, no. 835–849 (2024): e837, 10.1016/j.neuron.2023.11.023. PMC10939818 38134921

[73] M. R. Hayes , E. G. Mietlicki‐Baase , S. E. Kanoski , and B. C. De Jonghe , “Incretins and Amylin: Neuroendocrine Communication Between the Gut, Pancreas, and Brain in Control of Food Intake and Blood Glucose,” Annual Review of Nutrition 34 (2014): 237–260, 10.1146/annurev-nutr-071812-161201. PMC4458367 24819325

[74] D. I. Brierley , M. K. Holt , A. Singh , et al., “Central and Peripheral GLP‐1 Systems Independently Suppress Eating,” Nature Metabolism 3 (2021): 258–273, 10.1038/s42255-021-00344-4. PMC7116821 33589843

[75] A. Cauvin , P. Robberecht , P. De Neef , et al., “Properties and Distribution of Receptors for Pituitary Adenylate Cyclase Activating Peptide (PACAP) in Rat Brain and Spinal Cord,” Regulatory Peptides 35 (1991): 161–173, 10.1016/0167-0115(91)90478-y. 1661904

[76] Y. Masuo , N. Suzuki , H. Matsumoto , et al., “Regional Distribution of Pituitary Adenylate Cyclase Activating Polypeptide (PACAP) in the Rat Central Nervous System as Determined by Sandwich‐Enzyme Immunoassay,” Brain Research 602 (1993): 57–63, 10.1016/0006-8993(93)90241-e. 8095427

[77] K. Warfvinge and L. Edvinsson , “Cellular Distribution of PACAP‐38 and PACAP Receptors in the Rat Brain: Relation to Migraine Activated Regions,” Cephalalgia 40 (2020): 527–542, 10.1177/0333102419893962. 31810401

[78] E. Farkas , A. Szilvasy‐Szabo , Y. Ruska , et al., “Distribution and Ultrastructural Localization of the Glucagon‐Like Peptide‐1 Receptor (GLP‐1R) in the Rat Brain,” Brain Structure & Function 226 (2021): 225–245, 10.1007/s00429-020-02189-1. 33341919 PMC7817608

[79] G. Gu , B. Roland , K. Tomaselli , C. S. Dolman , C. Lowe , and J. S. Heilig , “Glucagon‐Like Peptide‐1 in the Rat Brain: Distribution of Expression and Functional Implication,” Journal of Comparative Neurology 521 (2013): 2235–2261, 10.1002/cne.23282. 23238833

[80] T. Gupta , M. Kaur , D. Shekhawat , R. Aggarwal , N. Nanda , and D. Sahni , “Investigating the Glucagon‐Like Peptide‐1 and Its Receptor in Human Brain: Distribution of Expression, Functional Implications, Age‐Related Changes and Species Specific Characteristics,” Basic and Clinical Neuroscience 14 (2023): 341–353, 10.32598/bcn.2021.2554.2. 38077175 PMC10700809

[81] S. E. Kanoski , M. R. Hayes , and K. P. Skibicka , “GLP‐1 and Weight Loss: Unraveling the Diverse Neural Circuitry,” American Journal of Physiology. Regulatory, Integrative and Comparative Physiology 310 (2016): R885–R895, 10.1152/ajpregu.00520.2015. 27030669 PMC4888559

[82] G. Sorensen , I. A. Reddy , P. Weikop , et al., “The Glucagon‐Like Peptide 1 (GLP‐1) Receptor Agonist Exendin‐4 Reduces Cocaine Self‐Administration in Mice,” Physiology & Behavior 149 (2015): 262–268, 10.1016/j.physbeh.2015.06.013. 26072178 PMC4668599

